# Spoligotype analysis of *Mycobacterium bovis* isolates from cattle and assessment of zoonotic TB transmission among individuals working in bovine TB‐infected dairy farms in Ethiopia

**DOI:** 10.1111/zph.12955

**Published:** 2022-05-18

**Authors:** Gizat Almaw, Adane Mihret, Tamrat Abebe, Gobena Ameni, Balako Gumi, Abebe Olani, Mekdes Tamiru, Tafesse Koran, Abde Aliy, Melaku Sombo, Sosina Ayalew, Adem Yesuf, Hawult Taye, James L. N. Wood, Stefan Berg, the ETHICOBOTS consortium, Abraham Aseffa, Bamlak Tessema, Bizuneh Belachew, Eshcolewyene Fekadu, Fantanesh Melese, Gizachew Gemechu, Rea Tschopp, Shewit Haile, Tsegaye Hailu, Adam Bekele, Chilot Yirga, Mulualem Ambaw, Tadele Mamo, Tesfaye Solomon, Tilaye Teklewold, Solomon Gebre, Getachew Gari, Mesfin Sahle, Asegedech Sirak, Getnet Mekonnen, Sintayehu Guta, James Wood, Andrew Conlan, Alan Clarke, Henrietta L. Moore, Catherine Hodge, R. Glyn Hewinson, Martin Vordermeier, Javier Nunez‐Garcia, Berecha Bayissa, Aboma Zewude, Adane Worku, Lemma Terfassa, Mahlet Chanyalew, Temesgen Mohammed, Yemisrach Zeleke

**Affiliations:** ^1^ National Animal Health Diagnostic and Investigation Center Sebeta Ethiopia; ^2^ Department of Microbiology, Immunology and Parasitology, College of Health Sciences Addis Ababa University Addis Ababa Ethiopia; ^3^ Armauer Hansen Research Institute Addis Ababa Ethiopia; ^4^ Aklilu Lemma Institute of Pathobiology Addis Ababa University Addis Ababa Ethiopia; ^5^ Department of Veterinary Medicine, College of Food and Agriculture United Arab Emirates University Al Ain United Arab Emirates; ^6^ Disease Dynamics Unit, Department of Veterinary Medicine University of Cambridge Cambridge UK; ^7^ Bacteriology Department Animal and Plant Health Agency Weybridge UK

**Keywords:** Central Ethiopia, dairy farms, *Mycobacterium bovis*, spoligotyping, zoonosis

## Abstract

Bovine tuberculosis (bTB) is a disease with impact on dairy productivity, as well as having the potential for zoonotic transmission. Understanding the genetic diversity of the disease agent *Mycobacterium bovis* is important for identifying its routes of transmission. Here we investigated the level of genetic diversity of *M. bovis* isolates and assessed the zoonotic potential in risk groups of people working in bTB‐infected dairy farms in central Ethiopia. *M. bovis* was isolated and spoligotyped from tissue lesions collected from slaughtered cattle as well as from raw milk collected from bTB positive cows in dairy farms from six urban areas of central Ethiopia. From consented dairy farm workers, knowledge and practices related to zoonotic TB transmission, together with demographic and clinical information, was collected through interviews. Sputum or Fine Needle Aspirate (FNA) samples were collected from suspected TB cases. Spoligotyping of 55 *M. bovis* isolates that originated either from cattle tissues with tuberculous lesion or from raw milk revealed seven spoligotype patterns where SB1176 was the most prevalent type (47.3%). Most isolates (89.1%) were of the *M. bovis* African 2 clonal complex. All sputum and FNA samples from 41 dairy farm workers with symptoms of TB were culture negative for any mycobacteria. Among the 41 TB suspected farm workers, 61% did not know about bTB in cattle and its zoonotic potential, and over two‐third of these workers practiced raw milk consumption. Our spoligotype analysis suggests a wider transmission of a single spoligotype in the study area. The results reported here may be useful in guiding future work to identify the source and direction of bTB transmission and hence design of a control strategy. Isolation of *M. bovis* from milk, knowledge gap on zoonotic TB and practice of consumption of raw milk in the study population showed potential risk for zoonotic transmission.

**Impacts**

- The study identified high level of genetic diversity of *Mycobacterium bovis* isolates in central Ethiopia indicating different sources of infection in an uncontrolled setting.
- Isolation of *M. bovis* from milk and common practice of raw milk consumption showed potential risk for zoonotic transmission in the study population.
- The lack of knowledge about zoonotic TB identified among risk groups working on bTB‐infected dairy farms highlights the importance of risk awareness for zoonotic diseases.

## 1 INTRODUCTION

*Mycobacterium bovis*, the causative agent of bovine tuberculosis (bTB), is a zoonotic bacterium of the *Mycobacterium tuberculosis* complex (MTBC) with a wide host range causing tuberculosis (TB) in humans and animals (Smith et al., 2006). In developed countries the bTB prevalence has been drastically reduced due to control programmes, mainly through test and slaughter of infected cattle together with milk pasteurization. In Africa, the disease is endemic, and this has economic implications for the growth of the livestock sector, especially in the intensive dairy sector where risk of transmission is often higher (Ameni et al., 2006) and poses the risk of zoonotic TB (zTB) transmission. zTB most often refers to human infection with *M. bovis* of animal origin (WHO, FAO and OIE, 2019) and it has been estimated to contribute to about 2.8% of all human TB cases in the African population (Müller et al., 2013). In Ethiopia, the demand for milk is expanding rapidly due to increased urbanization and population pressure; Ethiopia is the second most populous country in Africa with an estimated population of over 114 million people (UN, 2019). Most of the milk in the Ethiopian market system is not sold through the large dairies where pasteurization is practiced but sold direct to the consumers from smallholder farmers in an unpasteurized form. This increases the risk of zoonotic diseases transmission, including *M. bovis*, as a significant part of the population do not boil the milk but drink it raw (Deneke et al., 2022). In Ethiopia, although limited human studies on zTB prevalence have been conducted, the largest study conducted showed a relatively low prevalence of 0.4% *M. bovis* in the human population (Firdessa et al., 2013). However, it would be difficult to rule out the zoonotic significance given that few studies have been conducted in high‐risk populations. The low contribution of *M. bovis* in human TB in Ethiopia could be, among other things, due to lack of risk‐based surveillance such as people working in bTB‐infected dairy farms. In a direct contrast to this, tuberculin skin‐testing surveys over the last two decades have shown that bTB remains high in cattle, particularly in central Ethiopia with herd prevalence ranging between 50%–60% (Sibhat et al., 2017; Almaw et al., 2021), which is likely due to lack of a national control programme. As a comparison and argument for further exploring zTB in Ethiopia, it is worth mentioning that similar bTB prevalence in cattle in England and Wales in the 1940s, to the current bTB burden in Ethiopian cattle, was suggested to contribute to 5–6% zTB among all human TB cases in the two countries during that time (Hardie and Watson, 1992).

Any control intervention of bTB needs a thorough understanding of the genetic diversity of *M. bovis* and hence the transmission dynamics within herds, between herds and regions, and between farmed animals and wildlife, but also for tracing the origin of zTB cases (Machado et al., 2018). One of the most common epidemiological molecular typing methods applied to *M. bovis* is spoligotyping. While bTB is known to be highly prevalent in central Ethiopia, based on tuberculin skin test surveys, the information on molecular typing of *M. bovis* isolates in this region is limited (Sibhat et al., 2017; Romha et al., 2018). In addition, successful attempts to isolate *M. bovis* from milk have been rare in Ethiopia, so there is a need for better understanding of the prevalence and type of *M. bovis* in milk as consumption of raw milk and traditional fermented milk (such as the yoghurt ‘*Ergo*’) are common practices in Ethiopia. Here we aimed to examine the levels of genetic diversity of *M. bovis* isolates from tuberculous lesions and raw milk from cattle and assess the zoonotic potential in risk groups of people working in bTB‐infected dairy farms in central Ethiopia.

## 2 MATERIALS AND METHODS

### 2.1 Study design and study sites

**FIGURE 1:**
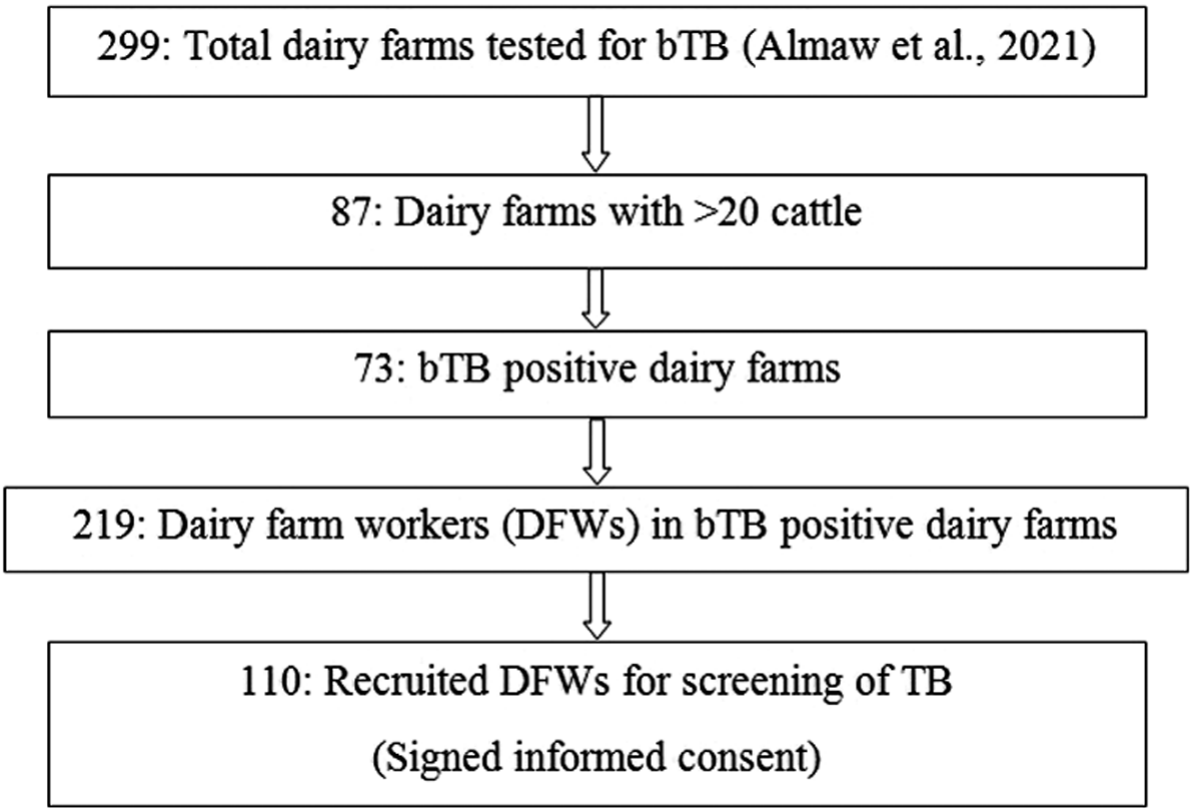
Flow chart of recruitment of dairy farm workers for active zTB surveillance

The cattle study included post‐mortem examination of cattle recruited at slaughterhouses and at dairy farms. It was a cross sectional study conducted from August 2018 to July 2019 at two slaughterhouses located in Addis Ababa city and in Sululta town northwest of Addis Ababa, in central Ethiopia. In addition, tuberculin skin test positive cattle, removed (voluntarily) from bTB‐infected dairy farms in Bishoftu and Holeta towns, two urban areas of central Ethiopia, were examined post‐mortem for detection of tuberculous lesions and for isolation of mycobacteria. All cattle examined in this study were considered as dairy cattle of the Holstein‐Frisian breed, or crosses thereof with the local zebu breeds. The zTB study was also a cross sectional study where dairy farm workers (DFW) were screened for active cases of TB and the recruitment procedure is described in Figure 1. Sampling took place between January and October 2020 and were based on the 299 dairy farms included in the bTB prevalence study described by Almaw et al. (2021), that covered six urban areas of central Ethiopia (Addis Ababa city, and Sebeta, Holeta, Sululta, Sendafa and Bishoftu towns). In the latter study, cattle were screened for bTB using the Single Intradermal Cervical Comparative Tuberculin (SICCT) test and interpreted according to OIE manual (OIE, 2018). Only the larger dairy farms (>20 cattle) that were positive for bTB (at least one reactor animal in the herd) were selected for recruitment of DFWs in the present zTB study. A DFW was defined as an individual (employee, family member or owner) of a selected dairy farm and was invited to participate for the active case detection of TB and those who provided informed consent were enrolled in this study.

### 2.2 Ante‐ and post‐mortem examination and milk sample collection

Addis Ababa Abattoir Enterprise and Sululta Cooperatives Abattoir were selected as study sites for tuberculous lesion detection in cattle based on their large number of daily slaughter and proximity to the laboratory at National Animal Health Diagnostic and Investigation Center (NAHDIC) for immediate transportation of specimens. Ante‐mortem examination recorded animal ID, sex, breed, origin and clinical signs of disease (if any) and a detailed post‐mortem examination of visceral organs and lymph nodes was conducted following standard procedures (Gracey et al., 1999). When a bTB‐like lesion was detected, a sample was taken and transported on ice to the Bio Safety Laboratory 3 (BSL3) at NAHDIC for processing and culturing of mycobacteria. Raw milk was collected for processing and culturing of mycobacteria from tuberculin SICCT test positive cows randomly selected among the 299 farms (Almaw et al., 2021).

### 2.3 Socio‐demographic and clinical data collection

DFWs recruited with written consent were screened by clinician for symptoms of pulmonary TB and TB lymphadenitis (TBLN) and the Ethiopian National Algorithm, MoH (2008), was used for recruitment TB patients. Demographic and clinical information was collected from each TB suspected individual through an interview using a questionnaire (Table S1). Data was also collected on potential risk factors such as consumption of raw milk and meat, occupation, physical animal contact etc. Sputum samples were collected from suspected TB patients using sterile 50 ml falcon tube. Fine needle aspirate (FNA) was collected aseptically from enlarged cervical lymph nodes by clinician and used for culture. Sputum and FNA samples were transported on ice to the TB laboratory at Armauer Hansen Research Institute (AHRI) in Addis Ababa. Culturing of mycobacteria from human specimens was conducted following previous protocols by Firdessa et al. (2013).

### 2.4 Isolation of *Mycobacterium bovis* from tuberculous lesions

Lymph nodes and visceral organs were screened for firm, hard nodule, purulent, caseous or calcified suspect tuberculous lesions (Gracey et al., 1999) and collected for isolation of mycobacteria. Visceral organs including lung lobes, thoracic membrane, heart, spleen, liver, intestines, kidneys, abdominal membrane, mammary gland (cows) and whole carcass were examined. Lymph nodes including parotid, mandibular, retropharyngeal, tracheal (apical), bronchial, mediastinal and carcass lymph nodes (axillary, pre‐scapular, pre‐femoral, popliteal) were examined following the standard procedure (Gracey et al., 1999).

Animal specimens were transported on ice to NAHDIC BSL 3 Zoonotic Laboratory and processed for mycobacterial culturing following standard procedures described elsewhere (WHO, 1998; Biffa et al., 2010). Duplicate egg‐based LJ medium (Löwenstein‐Jensen TB Medium Base, Sigma Aldrich) supplemented with 0.75% glycerol or 0.4% pyruvate was used, two media that favours growth of *M. tuberculosis* and *M. bovis*, respectively. The slants were incubated at 37°C and observed daily for growth of colonies during the first week, then on a weekly basis. Slants with no growth at week 8 were considered as negative.

### 2.5 *Mycobacterium bovis* isolation from raw milk

Thirty to fifty ml of raw milk was collected from bTB positive cows and transported at 4°C to NAHDIC BSL 3 Zoonotic Laboratory where it was processed for mycobacterial culturing immediately, or if delayed stored at −80°C for a maximum of 90 days. Mycobacterial culturing from milk was performed by following the methods described by Zumarraga et al. (2012) and Medeiros et al. (2011). Briefly, raw milk was centrifuged at 4000 × *g* for 15 min and the pellet was re‐suspended in 10 ml 0.75% (w/v) hexadecylpyridium chloride (HPC) (Sigma Aldrich) and incubated for 5 h at room temperature for decontamination (Medeiros et al., 2011). The decontaminated milk was again centrifuged (4000 × *g*, 15 min), decanted and the pellet suspended in 2 ml PBS, of which 500 μl was inoculated in duplicate on two slants of LJ medium supplemented with 0.75% glycerol and 0.5% pyruvate (increased pyruvate concentration as compared to culture protocol for tuberculous lesions), respectively. Incubation and interpretation were the same as for culturing from tuberculous lesions.

### 2.6 Genomic DNA extraction and RD4 deletion typing

Culture positive samples identified as Acid Fast Bacilli (AFB) by Ziehl‐Neelsen staining were analysed by RD4 deletion typing (PCR) to determine the status of RD4 (Region of Difference 4), a deletion that defines *M. bovis*.

The analysis was carried out as previously described (Biffa et al., 2010; Firdessa et al., 2012). Briefly, three to four bacterial colonies were harvested from a medium slant, heat‐killed at 80°C for 1 hr, and subjected to DNA extraction (QIAamp DNA Mini Kit). Following DNA extraction, RD4 typing was performed, and the result visualized by agarose gel electrophoresis. A PCR product of size 446 bp was interpreted as ‘Deleted for RD4’ and hence as *M. bovis*.

### 2.7 Conventional spoligotyping

Spoligotyping was performed following the methodology described by Kamerbeek et al. (1997) and a non‐commercial spoligotyping membrane produced by the Animal & Plant Health Agency (United Kingdom) was used for hybridization and detection of spacers. The spoligotypes were identified by recording the presence or absence of spacer signals. Reference strains *M. tuberculosis* H37Rv and *M. bovis* AF2122/97 were included as controls. Generated spoligotype patterns were compared with the *M. bovis* spoligotyping database (https://www.mbovis.org/) for identification of Spoligotype Bovis (SB) numbers.

### 2.8 Data analysis

Data from questionnaires were curated and coded and descriptive statistics was used to summarize and calculate proportions. The association of potential risk factors with clinical characteristics typical for TB among DFWs was analysed by using univariable and multivariable logistic regression. All variables with a *p*‐value <.2 were selected for the multivariable logistic regression and a *p*‐value of <.05 was considered statistically significant. For the level of occupational exposure, classification described by Torres‐Gonzalez et al. (2013) was used: high‐direct contact with cattle in closed spaces (1); medium‐direct contact with cattle in open spaces (2); low‐no direct contact with livestock (3). In our study, veterinarians and milkers were classified as having high exposure (1), in the medium category were family members, guards and farm workers other than milking (2), and in the low exposure category (3) were farm owners and farm managers. R software was used for all statistical analysis using the R statistical language (R Core Team, 2018) and RStudio (RStudio Team, 2020).

## 3 RESULTS

### 3.1 Isolation of *Mycobacterium bovis* from tissue, milk and human specimen

Post‐mortem examinations of 827 dairy cattle from abattoirs and farms identified 76 cattle (9.2%) with suspect tuberculous lesions in one or more organs, from which a total of 137 specimens were collected and processed for isolation of mycobacteria by culture (Table 1). Whenever multiple lesions were encountered, more than one specimen per animal were taken. Culturing and molecular typing yielded 62 *M. bovis* isolates from the 137 tuberculous lesions (45.3%) and they originated from 42 cattle: single *M. bovis* isolates came from twenty‐four animals while 18 animals generated more than one isolate per animal. In case of multiple isolates from an animal, they were always shown to carry the same spoligotype. For isolation of mycobacteria from raw milk, 490 composite milk samples were collected, processed and cultured for isolation of mycobacteria (Table 1) from 975 milking cows, which were tuberculin SICCT positive (37.8%, *n* = 2582) among 299 dairy farms previously tested in central Ethiopia (Almaw et al., 2021). Culturing yielded 11 *M. bovis* isolates, which corresponds to 2.2% of the processed milk samples; no other type of mycobacteria was isolated from milk and no post‐mortem examination was conducted on cows from which milk was collected. A total of 44 human specimens (sputum = 41; FNA = 3) were collected from 41 TB suspected individuals but attempts to culture from these samples resulted in no mycobacterial isolates.

**TABLE 1:** *Mycobacterium bovis* isolates from tuberculous lesions tissue lesion and raw milk

Sample type		Abattoir/farm location						
		Sululta	Addis Ababa	Bishoftu	Holeta	Sebeta	Sendafa	Total (%)
Tissue TB lesion	Number of animals examined by post‐mortem (a)^a^	451	350	11	15	–	–	827
	Number of animals with tuberculous lesion (b) (% = b/a*100)	29 (6.4)	27 (7.7)	9 (81.8)	11 (73.3)	–	–	76 (9.2)
	Number of animals with *M. bovis* isolated (c) (% = c/b*100)	16 (55.2)	10 (37)	6 (66.7)	10 (90.9)	–	–	42 (55.3)
Raw milk	Number of samples collected from bTB positive cows (d)	40	44	100	73	120	113	490
	Number of *M. bovis* isolated (e) (% = e/d*100)	0	0	2 (2)	2 (2.7)	7 (3.3)	0	11 (2.2)

^a^ Letters in bracket (a–e) show how the % in the table is calculated.

### 3.2 Spoligotype analysis

Out of the 73 *M. bovis* isolates that originated from cattle tissues and milk, spoligotype patterns were determined for 55 isolates (47 were from tissues and eight from milk, Table S2). Among the seven patterns identified, spoligotype SB1176 was the most prevalent type (47.3%), followed by SB0912 (14%), SB0134 (11%) and SB0133 (11%), while the least was SB2521 (2%), a single isolate (Table 2). SB1176 was distributed across all sites except Sendafa from where only milk samples were collected and no *M. bovis* was isolated.

**TABLE 2:** Geographic distribution of *Mycobacterium bovis* spoligotypes in this study

Spoligotype number	Study site						Total (%)
	Sululta	Addis Ababa	Bishoftu	Holeta	Sebeta	Sendafa	
SB1176	10	5	7 (^a^1)	1 (1)	3 (3)	0	26 (47.3)
SB0912	1	0	0	7	0	0	8 (14.5)
SB0133	3	2	0	0	1	0	6 (10.9)
SB0134	3	3	0	0	0	0	6 (10.9)
SB1521	1	0	0	2	1 (1)	0	4 (7.3)
SB1878	2	0	1 (1)	1 (1)	0	0	4 (7.3)
SB2521	0	1	0	0	0	0	1 (1.8)

^a^ Numbers in bracket represent isolates from milk, for example ‘7 (1)’ equals seven isolates of which one is from milk.

The two slaughterhouses in Sululta and Addis Ababa generated spoligotyped 32 *M. bovis* isolates that included all seven spoligotype patterns (Table 2) identified in this study. The remaining 23 isolates (15 isolates from tissue and 8 from milk) were of four patterns (SB1176, SB0912, SB1521, SB1878) and they originated from 10 dairy farms in Bishoftu, Holeta and Sebeta. In two of these farms, more than one spoligotype per farm were identified showing the presence of mixed infections at farm level.

### 3.3 Demographic and clinical characteristics of dairy farm workers

All DFWs (*n* = 219) from the 73 dairy farms with bTB reactor cattle were invited to participate in the survey for active case detection of TB and those who provided informed consent (*n* = 110) were enrolled in this study. Our post‐mortem and milk culture work confirmed *M. bovis* infection in cattle at 10 out of these 73 farms. Among the 110 DFWs who were screened for human TB, 41 had at least one of the six constitutional symptoms shown in Table 3 that are typical for TB (i.e. coughing sputum, coughing blood, chest pain, weight loss, fever and night sweats). None of the DFWs had signs of all six constitutional symptoms, but one patient had 5/6 symptoms, seven patients had 4/6 symptoms, 14 patients had 3/6 symptoms, 11 patients had 2/6, while eight patients had only 1/6 of the constitutional symptoms. Three DFWs had swollen nodes at their neck, a symptom typical for TB lymphadenitis, and they were sampled for FNA. Two DFWs had a history of TB.

**TABLE 3:** Demographic and clinical characteristics for TB suspected dairy farm workers

	Characteristic	Category	Frequency	%
Demographic	Sex	Female	8	19.5
		Male	33	80.5
	Age	≤20 years	8	19.5
		>20–≤45 years	21	51.2
		>45 years	12	29.3
	Education	Illiterate	15	36.5
		Primary	14	34.1
		Secondary	9	21.9
		College/University	3	7.3
	Marital status	Single	18	43.9
		Married	21	51.2
		Separated	0	0
		Divorced	1	2.4
		Widowed	1	2.4
Clinical	Currently coughing up sputum	Yes	20	48.8
	Currently coughing up blood	Yes	1	2.4
	Chest pain	Yes	12	29.3
	Weight loss	Yes	18	43.9
	Fever	Yes	23	56.1
	Night sweats	Yes	30	73.1
	Presence of swollen nodes at neck	Yes	3	7.3
	BCG vaccination	Yes	9	21.9
	History of TB	Yes	2	4.9

### 3.4 Assessment of knowledge and practices related to zoonotic TB and analysis of potential risk factors

The majority (61%) of the 41 TB suspected DFWs did not know about cattle TB (Table 4). Raw milk consumption was practiced by more than two thirds of the DFWs with symptoms (68.2%, Table 4). The practice of raw meat consumption among DFWs with symptoms was much higher (85.4%, Table 4) as compared to raw milk. More than two thirds of DFWs with symptoms did not think TB could be transmitted via raw or undercooked meat.

**TABLE 4:** Knowledge and practices on risk factors for zoonotic TB transmission

Practice/knowledge/risk factor	Category	Frequency	%
Consumption of raw milk	Yes	28	68.2
	No	13	31.7
Consumption of *Ergo* (traditional fermented milk)	Yes	31	75.6
	No	10	24.4
Do you think drinking raw milk or ergo can transfer TB from animals to humans?	Yes	21	51.2
	No	20	48.8
What is your main source of meat?	Home slaughter	3	7.3
	Butchery	16	39.0
	Communal slaughter (‘*Qircha*’)	9	22.0
	More than one source	13	31.7
Consumption of raw meat/kitfo/Dulet/Kurt	Yes	35	85.4
	No	6	14.6
Do you think eating raw meat/kitfo/Dulet/Kurt can transfer TB from animals to humans?	Yes	12	29.3
	No	29	70.7
Do you have close contact with cattle?	Yes	34	82.9
	No	7	17.1
Were there coughing cattle in your herd?	Yes	21	51.2
	No	20	48.8
Do you know about cattle TB?	Yes	16	39.0
	No	25	61.0
Occupation	a. Farm owner b. Veterinarian c. Guard/shepherd d. Farm worker (other than milking) e. Farm manager f. Family member g. Milker	3 1 10 9 1 2 15	7.3 2.4 24.4 21.9 2.4 5.0 36.6

A risk factor analysis was performed comparing DFWs with and without constitutional TB symptoms. Fourteen risk factors (sex, age, level of education, income level, raw milk consumption, Ergo (traditional fermented milk) consumption, knowledge about transmission of TB via raw milk or Ergo, raw meat consumption, knowledge about transmission of TB via raw or undercooked meat consumption, level of occupational exposure, close contact with cattle, coughing cattle presence at the farm, knowledge about cattle TB and BCG vaccination status) were considered and screened by univariable logistic regression analysis. Eight variables (age, income level, raw milk consumption, Ergo consumption, raw meat consumption, knowledge about transmission of TB via raw or undercooked meat consumption, close contact with cattle, BCG vaccination status) with p‐value of <.20 were selected for multivariable logistic regression analysis, but only age and income level were found to be significantly associated with symptoms typical for TB (Table 5).

**TABLE 5:** Significant risk factors in multiple logistic regression analysis associated with symptoms of TB

Risk factor	Total screened	Cases	Controls	OR (CI: 95%)	*p*‐value
Age					
Young (≤20 years)	17	8	9	2.01 (0.4–9.9)	0.37331
Adult (>20–≤45 years)	74	21	53	Reference	
Elder (>45 years)	19	12	7	1.7 (0.2–20.0)	0.01121
Income (Eth. Birr^a^)					
Very low (≤1000)	11	8	3	18.8 (16.9–29.8)	0.02306
Low (1001–2000)	60	26	34	3.3 (0.6–28.4)	0.21064
Medium (2001–3000)	21	4	21	1.2 (0.1–12)	0.88461
High (≥3001)	17	2	15	Reference	

^a^ Ethiopian birr.

## 4 DISCUSSION

In the present study we aimed to assess the genetic diversity of *M. bovis* isolated from cattle tissues with tuberculous lesions and from raw milk, as well as to explore the potential zoonotic risk of *M. bovis* transmission among DFWs from bTB positive dairy farms in central Ethiopia.

The 55 spoligotyped *M. bovis* isolates revealed seven patterns, where SB1176 was the most prevalent type (47.3%) and with a wider geographical distribution. In central and other parts of Ethiopia, Biffa et al. (2010) and Firdessa et al. (2012) also recorded SB1176 as a predominant spoligotype suggesting this type being widely distributed in Ethiopia and well established through the free movement of cattle in the region. There was also a more localized strain ‐ SB0912; the second prevalent spoligotype (14.5%) ‐ where all except one were isolated from a single farm. The East African dominant spoligotype (SB0133), which has been isolated from cattle in Uganda and Tanzania (Oloya et al., 2007; Berg et al., 2011, Romha et al., 2018) was also recorded in this study (10.9%). This suggests a wider distribution, most possibly due to the movement of livestock and wild animals. Cross border illegal livestock trade is a common practice in the East Africa region.

Bovine TB was first recorded in Ethiopian cattle around 1970 (FAO, 1967; Hailemariam, 1975) but has likely been endemic for a much longer time. Phylogeographic analysis of a global collection of 3364 whole‐genome sequences from *M. bovis* and *M. caprae* have even suggested that *M. bovis* likely originated in East Africa (Loiseau et al., 2020). *M. bovis* can be divided into several lineages, or clonal complexes, where each complex is genetically marked by the lack of specific spacers in their spoligotypes. All typed isolates of this study lacked spacers that are markers for *M. bovis* (Smith et al., 2006) and most (89.1%) of the isolates did also lack spacers 3–7 that marks the *M. bovis* African 2 clonal complex (Af2). This complex is believed to be restricted to East Africa in comparison to the African 1 clonal complex (Af1) that seems confined to West Africa (Berg et al., 2011; Müller et al., 2009). No isolate of Af1 was identified in this Ethiopian study but the remaining six isolates (10.9%) were of spoligotype SB0134 and likely members of the Eu3 clonal complex, which is prevalent in central Europe (Branger et al., 2020; Loiseau et al., 2020) but also present in Eritrea (Gebremariam et al., 2018).

Among 10 herds, five shared the same spoligotype (SB1176) though located in different locations (not adjacent herds) and this needs to be supported by epidemiological data to prove herd to herd transmission. Two herds were identified with more than one spoligotype (within herd diversity) and one herd had a spoligotype not detected in the other herds. The underlying reasons for this diversity might result from the presence of multiple sources of infection particularly for between herd diversity and/or a higher mutation rate (within herd diversity), although *M. bovis* has slow mutation rate (Crispell et al., 2017).

According to Grange and Yates (1994), tuberculosis in cattle is principally a pulmonary disease and only 1% of the tuberculous cows excrete tubercle bacilli in their milk. In this high bTB burden region of Ethiopia, we were able to isolate *M. bovis* from raw milk from 2.2% of cows that tested positive for bTB, signifying its zoonotic potential. Optimization of the LJ culturing medium with 0.5% pyruvate and a less harsh decontamination protocol, that is 0.75% (w/v) HPC instead of 4% NaOH, is likely to have helped improve the isolation rate of *M. bovis* from milk. According to a review of this topic in Ethiopia by Mengistu and Enquselassie (2014), the average percent of *M. bovis* detected from milk was 1.1% and for other *Mycobacterium* species it was 7.1%. Elias et al. (2008) detected 5 *M. bovis* from 141 reactor cow's raw milk (3.5%) in Addis Ababa using 2% NaOH for decontamination and they used biochemical tests for species confirmation. Ameni et al. (2003) used 3% NaOH and did not detect *M. bovis* from 24 raw milk samples that were collected from reactor cows in central Ethiopia. Kazwala et al. (1998) processed a large number of raw milk samples (*n* = 805) in Tanzania using 4% NaOH and detected 2 *M. bovis* (0.2%). The stage of the disease, the decontamination method used, and the characterization method (biochemical tests or by PCR) could be potential sources for variable detection rates in milk. Unfortunately, in this study we did not have the opportunity to perform post‐mortem on these cows shedding *M. bovis* in milk. This could have enabled us to examine the correlation between *M. bovis* excretion in milk and the detection of tuberculous lesions especially in udder and mammary lymph nodes. Spoligotypes of *M. bovis* isolates from milk in this study (SB1176, SB1521 and SB1878) were also recorded among isolates from tissues and suggest the existence of such correlation.

We performed an active TB surveillance among farm workers in 73 bTB positive dairy farms and 41 responded having at least one of six recorded constitutional symptoms that are typical for TB. In fact, eight of these DFWs had symptoms of at least four of these six possible signs of TB. However, sampling of sputa or FNA from these suspected cases yielded no *M. bovis* or any other mycobacterial cultures. This study was an active case surveillance where we went to the community for screening of TB from the apparently healthy population and this might decrease chance of detecting cases in advanced stages. In hospital‐based studies, patients themselves show up at the health facilities, because they are ill and the disease would likely be in a more advanced stage. Samples from such patients are likely to become culture positive.

Still, it could be misleading to conclude that *M. bovis* has no significant zoonotic contribution among DFWs in central Ethiopia, as culture is less sensitive in paucibacilliary specimens. The decontamination process may kill up to 60% of mycobacteria while processing a specimen (Etchechoury et al., 2010). We also investigated their knowledge and practices related to zoonotic TB and, among TB suspected individuals, 61% did not know about cattle TB. This figure was high when compared to other African countries, such as Nigeria, where 40% did not know about zoonotic TB (Adesokan et al., 2018). In Eastern Ethiopia, Kemal et al. (2019) recorded a similar finding; only 33% had the knowledge of, or had heard about bTB, and 23% of the respondents were aware of the zoonotic importance of this disease.

## 5 CONCLUSION

This study isolated 73 *M. bovis* strains from tissues and milk sampled from dairy cattle from central Ethiopia and we recorded seven spoligotypes of which all were previously reported from Ethiopia. The most prevalent spoligotype detected in our sample was SB1176. A knowledge gap on bTB and practice of raw milk consumption among the investigated farm workers, in combination with *M. bovis* isolation from milk of SICCT test positive cattle, showed that there is a clear potential risk for zoonotic transmission and a need for further investigation. With tools such as whole‐genome sequencing, new epidemiological studies can be performed, also in Ethiopia, to improve our understanding of the *M. bovis* population structure, its routes of transmission and zoonotic impact—information all important for designing control strategies in countries endemic for bTB.

## AUTHOR CONTRIBUTIONS

James L. N. Wood, Stefan Berg, Gobena Ameni and Adane Mihret conceptualized the article; Gizat Almaw analysed the data; Gizat Almaw, Abebe Olani, Abde Aliy, Mekdes Tamiru, Tafesse Koran, Melaku Sombo, Sosina Ayalew, Hawult Taye, Adem Yesuf involved in the investigation; Gizat Almaw, Stefan Berg, Adane Mihret, James L. N. Wood, Gobena Ameni involved in the methodology; Gizat Almaw wrote the original draft; Gizat Almaw, Stefan Berg, Adane Mihret, Tamrat Abebe, Gobena Ameni, Balako Gumi, James L. N. Wood reviewed and edited the article.

## CONFLICT OF INTEREST

The authors declare that they have no conflict of interests.

## ETHICAL APPROVAL

The study was reviewed and approved by Institutional Review Board (Protocol number: 073/19/DMIP) of College of Health Sciences, Addis Ababa University, Ethiopia.

## Supporting information


Table S1

Table S2

## Data Availability

The data that supports the findings of this study is available in the supplementary material of this article. Data on human subjects can be available on request due to privacy.
