# Supplementary material for: Spoligotype analysis of Mycobacterium bovis isolates from cattle and assessment of zoonotic TB transmission among individuals working in bovine TB-infected dairy farms in Ethiopia
Source: Zoonoses Public Health. Author manuscript; Available in PMC 2022 Oct 14. (PMC9544066; doi:10.1111/zph.12955)
Supplement: supplementary material 1 [file NIHMS1840861-supplement-supplementary_material_1.doc]

**Active Case Detection of TB at Dairy Farms**

**Part I. Socio-Demographic Characteristics**

GA

**Name________________________________________Patient Code________________Phone no._________________**

**Address: Region_____________________Zone/subcity___________________Woreda/town________________Kebele_________**

**Farm Name_____________________________________Farm Code_________________________Date_____________________**

| **S/N** | **Question** | **Response (circle the answer)** |
| --- | --- | --- |
| HAC1 | Sex | a. Male  b. Female |
| HAC2 | Age | ----------Years |
| HAC3 | Educational status | a. Illiterate (Unable to read and write)  b. Primary  c. Secondary  d. College/University |
| HAC4 | Monthly income | a. ≤1000 Eth. birr  b. 1001-2000 Eth. birr  c. 2001-3000 Eth. birr  d. ≥ 3001 Eth. birr |
| HAC5 | Marital status | a. single |
|  |  | b. married |
|  |  | c. separated |
|  |  | d. divorced |
|  |  | e. widowed |

**PART-II. Data on Potential Risk factors**

| **S/N** | **Questions** | **Response (circle the answer)** |
| --- | --- | --- |
| HAC6 | Consumption of raw milk | 1. Yes, if yes how often  a. regularly (daily)  b. twice per week  c. once per week  d. only once in life-time  e. other (mention)____________  2. No |
| HAC7 | Consumption of Ergo | 1. Yes, if yes how often  a. twice per week  b. once per week  c. twice per month  d. only once in life-time  e. other (mention)_____________  2. No |
| HAC8 | Do you think drinking raw milk or ergo can transfer TB from animals to humans? | 1. Yes  2. No |
| HAC9 | What is your main source of meat? | 1. home slaughter  2. buchery  3. communal slaughter (Qircha)  4. all |
| HAC10 | Consumption of raw meat/kitfo/dulet/kurt | 1. Yes, if yes how often  a. twice per week  b. once per week  c. twice per month  d. only once in life-time  e. other(mention)_______________  2. No |
| HAC11 | Do you think drinking raw meat/kitfo/dulet/kurt can transfer TB from animals to humans? | 1. Yes  2. No |
| HAC12 | Your occupation | a. farm owner/dairy or other animal farmer  b. veterinarian  c. guard/shepherd  d. farm worker (other than milking)  e. farm manager  f. family member  g. milker |
| HAC13 | Do you have close contact with cattle? | 1. Yes  2. No |
| HAC14 | Were there coughing cattle in your herd? | 1. Yes  2. No |
| HAC15 | Do you know about cattle TB? | 1. Yes  2. No |

**PART-III. Clinical information**

| **S/N** | **Signs & symptoms** | **Response (circle the answer)** |
| --- | --- | --- |
| HAC16 | Currently coughing up sputum or phlegm | 1. Yes  2. No |
| HAC17 | Currently coughing up blood | 1. Yes  2. No |
| HAC18 | Currently have chest pain | 1. Yes  2. No |
| HAC19 | Weight loss (in the last month) | 1. Yes  2. No |
| HAC20 | Fever | 1. Yes  2. No |
| HAC21 | Night sweats | 1. Yes  2. No |
| HAC22 | Loss of appetite | 1. Yes  2. No |
| HAC23 | Weakness | 1. Yes  2. No |
| HAC24 | Swelling around your neck (excluding goiter, trauma etc...) | 1. Yes  2. No |
| HAC25 | If yes to question HAC24, feel pain on your swelling | 1. Yes  2. No |
|  | **Characteristic of swelling (physical examination)** | |
| HAC26 | Duration of neck swelling | a. 0-4 weeks  b. >4-12 weeks  c. >12-52 weeks  d. >52 weeks |
| HAC27 | Perceived increase rate | a. slow  b. moderate  c. fast |
| HAC28 | Pain swelling | a. painless  b. painful |
| HAC29 | Location of nodes | a. unilateral  b. bilateral |
| HAC30 | Tenderness of node | a. non tender  b. tender |
| HAC31 | Number of nodes | a. single  b. few (2-4)  c. multiple (>=5) |
| HAC32 | Mobility of node | a. non mobile  b. mobile |
| HAC33 | Type | a. soft |
|  |  | b. discrete |
|  |  | c. matted |
|  |  | d. hard |
|  |  | e. firm |
|  |  | f. drainage sinus |

**Past Medical History of Tuberculosis**

| **S/N** | **Signs & symptoms** | **Response (circle the answer)** |
| --- | --- | --- |
| HAC34 | BCG vaccination | 1. Yes  2. No  3. Do not know |
| HAC35 | History of TB | 1. Yes  2. No  3. Do not know |
| HAC36 | If yes when? | 1. before 6 months |
|  |  | 1. before 1 year |
|  |  | 1. before 2 years |
|  |  | 1. before 5 years |
|  |  | 1. Other (mention)__________ |
| HAC37 | Have you been treated with anti-TB drugs? | 1. Yes  2. No |
| HAC38 | If yes to question HAC36, for how long did you take the treatment? | _____________/months/weeks/ |
| HAC39 | If yes to question Q HAC36, what was your treatment outcome? | a. cured  b. completed  c. default  d. failure  e. relapse  f. I do not know |
